# Efficacy and Safety of Sipjeondaebo-Tang for Anorexia in Patients with Cancer: A Pilot, Randomized, Double-Blind, Placebo-Controlled Trial

**DOI:** 10.1155/2017/8780325

**Published:** 2017-12-26

**Authors:** Chunhoo Cheon, Jeong-Eun Yoo, Hwa-Seung Yoo, Chong-Kwan Cho, Sohyeon Kang, Mia Kim, Bo-Hyoung Jang, Yong-Cheol Shin, Seong-Gyu Ko

**Affiliations:** ^1^Department of Preventive Medicine, Korean Medical College, Kyung Hee University, Seoul, Republic of Korea; ^2^Dunsan Korean Medicine Hospital of Daejeon University, Daejeon, Republic of Korea; ^3^Department of Cardiovascular and Neurologic Disease (Stroke Center), College of Korean Medicine, Kyung Hee University, Seoul, Republic of Korea

## Abstract

**Background:**

Anorexia occurs in about half of cancer patients and is associated with high mortality rate. However, safe and long-term use of anorexia treatment is still an unmet need.

**Objective:**

The purpose of the present study was to examine the feasibility of Sipjeondaebo-tang (Juzen-taiho-to, Shi-Quan-Da-Bu-Tang) for cancer-related anorexia.

**Methods:**

A total of 32 participants with cancer anorexia were randomized to either Sipjeondaebo-tang group or placebo group. Participants were given 3 g of Sipjeondaebo-tang or placebo 3 times a day for 4 weeks. The primary outcome was a change in the Anorexia/Cachexia Subscale of Functional Assessment of Anorexia/Cachexia Therapy (FAACT). The secondary outcomes included Visual Analogue Scale (VAS) of anorexia, FAACT scale, and laboratory tests.

**Results:**

Anorexia and quality of life measured by FAACT and VAS were improved after 4 weeks of Sipjeondaebo-tang treatment. However, there was no significant difference between changes of Sipjeondaebo-tang group and placebo group.

**Conclusions:**

Sipjeondaebo-tang appears to have potential benefit for anorexia management in patients with cancer. Further large-scale studies are needed to ensure the efficacy.

**Trial Registration:**

This trial is registered with ClinicalTrials.gov NCT02468141.

## 1. Introduction

Anorexia is associated with high mortality rate in cancer patients, reduction in the effectiveness of anticancer therapy, reduced ability to perform activities of daily living, and lower quality of life in physical, mental, and social functions [1]. It occurs in about half of cancer patients and is known to have the highest frequency in lung and upper digestive tract cancer [2]. It is important to properly manage anorexia, because cancer-related anorexia negatively affects response to chemotherapy and radiotherapy [3].

There were case studies on the improvement of anorexia in cancer patients using acupuncture [4] and acupressure [5], and there was a clinical study of moxibustion treatment for anorexia in patients with thyroid cancer [6]. Steroids, megestrol acetate, prokinetic agents (metoclopramide), ghrelin, melatonin, and so on are administered to treat cancerous anorexia state of cancer patients. Steroids can improve quality of life and appetite, but can only be used for a short period of time due to limitations of metabolism and infectious side effects. Megestrol acetate improves appetite but also has potential side effects such as water retention and vein embolism [7]. In addition, prokinetic agents such as metoclopramide improve chronic nausea, but there is no apparently proven effect on improving appetite [8]. It has been reported in a small number of cancer patients that ghrelin stimulates appetite and food intake [9].

Sipjeondaebo-tang (SJDBT, also known as Juzen-taiho-to in Japanese and Shi-Quan-Da-Bu-Tang in Chinese), one of the most commonly used traditional herbal medicines in Korea, is prescribed for patients with deficiency syndrome, suffering from anemia, fatigue, and anorexia [10, 11]. SJDBT also has been known to have anticancer effects [12], and in vivo study provided evidence on that SJDBT may be useful for patients with cancer associated anorexia [13].

The purpose of this study was to evaluate the effect and safety of SJDBT for anorexia in cancer patients by comparing the experimental group to placebo group using the Functional Assessment of Anorexia and Cachexia Treatment (FAACT; Anorexia/Cachexia Subscale [ACS], Functional Assessment of Cancer Therapy-General [FACT-G]), anorexia Visual Analogue Scale (VAS), weight, body mass index (BMI), adrenocorticotropic hormone (ACTH), and cortisol values.

## 2. Materials and Methods

### 2.1. Study Design

**Figure 1:**
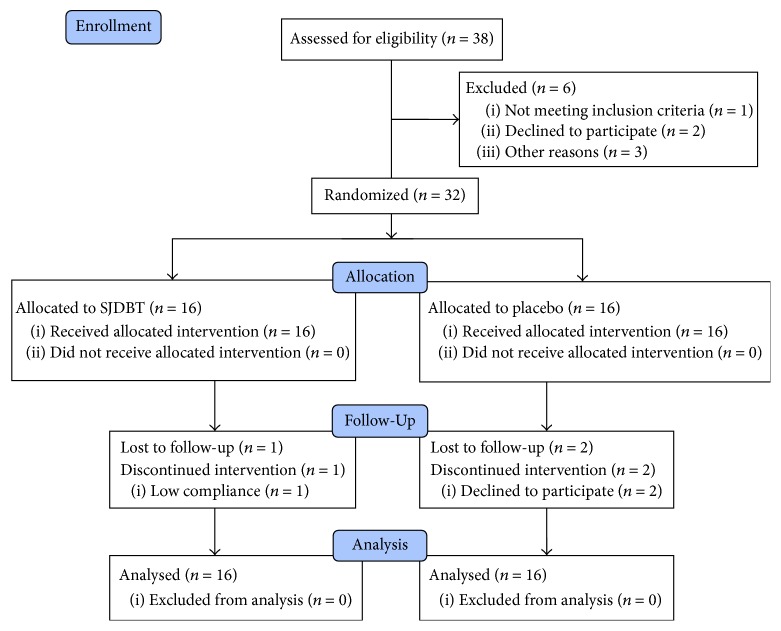
Participants flow chart.

A randomized, double-blind, placebo-controlled trial was conducted at the Dunsan Korean Medicine Hospital of Daejeon University in Daejeon, Republic of Korea. The Institutional Review Board of the Dunsan Korean Medicine Hospital of Daejeon University approved the study (reference DJDSKH-15-03-2 (Ver. 2.0)). The study protocol of this study has already been published as a protocol paper and also registered in ClinicalTrials.gov (NCT02468141) prior to the completion of the clinical trial. We followed the methods of Cheon et al. 2016 [14]. The process actually performed was described in the present report. Written informed consent was obtained from each participant prior to the study procedures. Participants who fulfil the eligibility criteria were enrolled. The enrolled participants were randomly assigned to two parallel groups: the SJDBT group and placebo group with the allocation ratio of 1 : 1. Each participant was assessed for signs and symptoms of anorexia during the trial period. The participants flow chart is shown in Figure 1.

This study was a pilot study that examines the feasibility for a full randomized clinical trial of SJDBT for treating cancer-related anorexia and determines the effect size for further large-scale studies. Thus, a total of 32 participants were recruited for the study. Sixteen participants were allocated to the SJDBT group and another sixteen to the placebo group.

SJDBT and placebo were produced and packaged by Hanpoong Pharm and Foods Co., Ltd., the pharmaceutical company, certified GMP (Good Manufacturing Practice) by the Ministry of Food and Drug Safety of Korea. SJDBT includes* Cinnamomi Cortex* (1.00 g),* Paeoniae Radix* (1.00 g),* Atractylodis Rhizoma Alba* (1.00 g),* Ginseng Radix Alba* (1.00 g),* Cnidii Rhizoma* (1.00 g),* Astragali Radix* (1.00 g),* Poria Sclerotium* (1.00 g),* Rehmanniae Radix Preparata* (1.00 g),* Angelicae Gigantis Radix* (1.00 g), and* Glycyrrhizae Radix* (0.5 g). These raw materials were extracted and concentrated to 3 grams for single dose. The placebo was made of lactose, corn starch, and caramel colouring, and it had similar appearance, shape, weight, taste, and colour to SJDBT. Investigational products were manufactured in accordance with the Korean herbal pharmacopoeia and the Korean pharmacopoeia [15, 16]. Brief description is as follows: Each herbal medicine of SJDBT and 10-fold volume of purified water are put into an extractor and extracted at 100°C for 3 hours. The extract is filtered using a microfilter with a size of 25 *μ*m, and the filtrate is concentrated under reduced pressure at 60°C or lower and dried to obtain dried extract.

The participants received SJDBT or a placebo drug for four weeks. They orally took 3 grams of granules with water three times a day after meals for 4 weeks. The participants were required to return the remaining drug for calculating compliance at visit 3 and visit 4. During the trial, the participants were prohibited to receive other treatments for anorexia. All treatments were documented on the case report form (CRF), including the drug name, the daily dose, the purpose of the drug use, the route of administration, and the start date and stop date.

### 2.2. Participants

Regardless of the type and stage of cancer, all patients with solid cancer complaining of anorexia have been subject to recruitment. Participants were recruited regardless of chemotherapy and radiotherapy. The inclusion and exclusion criteria are shown in Box 1.

### 2.3. Randomization and Blinding

Institute of Safety, Efficacy and Effectiveness Evaluation for Korean Medicine (ISEE) which was the Contract Research Organization (CRO) of the present study generated random numbers. Block randomization with block size of four was performed. The random numbers and randomization table were maintained by the ISEE during the research period in opaque and sealed envelopes. The opening procedure followed the Standard Operating Procedures (SOPs). A research assistant who was not involved in recruitment, intervention, or assessment of outcomes prepared the envelopes. Investigators responsible for recruitment and assessment were not allowed to take part in the group allocation. Therefore, all the investigators, monitors, and participants were blinded for assignment of the study drugs.

### 2.4. Efficacy Measurements

The change in the ACS of FAACT between the baseline (visit 2) and end of the study (visit 4) was used as the primary outcome measurement [17]. Secondary outcome measurements were as follows: the changes in the FACT-G [17], anorexia VAS, qi deficiency and blood deficiency scale [18], and clinical laboratory tests including ACTH, cortisol, ghrelin, IL-6, erythrocyte sedimentation rate (ESR), and c-reactive protein (CRP). Blood and urine specimens were collected for haematological test, biochemistry test, and urinalysis at visit 1, visit 2, and visit 4. The specimens were analysed in the clinical laboratory of trial institution.

### 2.5. Safety Measurement

At every visit, vital signs including blood pressure, pulse, respiration rate, and physical examination, haematologic test, biochemical test, and urine test related to safety assessment and any adverse events were documented in the CRFs.

### 2.6. Statistical Analysis

All statistical procedures were performed using the R statistical software (version 3.2.5; R Foundation for Statistical Computing, Vienna, Austria). A two-sided, 5% or lower *p* value was considered statistically significant. ITT (intention-to-treat, all randomly assigned participants) data set was applied for data analysis. The continuous variables were displayed as mean ± SD, and the categorical variables were displayed as *n* (%). The baseline characteristics were compared to see whether there is a significant difference between SJDBT group and placebo group by either an independent *t*-test for continuous variables including age, height, weight, BMI, FAACT scale, anorexia VAS, SBP, DBP, pulse, ACTH, cortisol, ghrelin, interleukin 6, ESR, and CRP or the *χ*^2^ test for the categorical data including gender, cancer type, and anticancer therapy. For the efficacy variables, the mean differences between the baseline and the end of the treatment were compared using an independent *t*-test. Two-way repeated measured analysis of variance was also used to determine differences between the groups and over time. The normality assumption was tested using Shapiro-Wilk test.

## 3. Results

Of all the 32 randomized participants, 16 participants (50%) took SJDBT and 16 participants (50%) took placebo. There were 14 thyroid cancer, 7 breast cancer, 4 lung cancer, 2 breast cancer, 2 colon cancer, and 2 cervical cancer participants. Of the total 32 participants, 3 participants received both chemotherapy and radiation therapy, 7 participants received chemotherapy only, 9 participants received radiotherapy only, and 13 participants did not receive both chemotherapy and radiation therapy. In SJDBT group, there was one participant lost to follow-up for low compliance. In placebo group, there were two participants lost to follow-up who withdrew the informed consent. No one dropped out before receiving the intervention. Thus, 16 participants treated with SJDBT and 16 participants treated with placebo were included in the ITT analyses. After 4 weeks, 15 participants completed the trial in the SJDBT group and 14 participants completed the trial in the placebo group. Participants flow chart is shown in Figure 1.

### 3.1. Baseline Characteristics

The baseline characteristics of the two groups were shown in Table 1. Baseline characteristics were similar in the two groups. There were no statistically significant differences between the SJDBT group and placebo group with respect to age, sex, height, weight, BMI, FAACT scale, anorexia VAS, vital signs, and laboratory tests (*p* > 0.05).

### 3.2. Primary Outcomes

#### 3.2.1. FAACT ACS

The mean FAACT ACS score at each time point is shown in Table 2. The changes in the Anorexia/Cachexia Subscale of FAACT between baseline and the end of study were −4.63 in SJDBT group and −2.75 in placebo group, but the changes of FAACT ACS score were not significantly different between the two groups (*p* = 0.245). In the within-group analysis, change in SJDBT group and placebo group showed significant difference (*p* < 0.001 and *p* = 0.025, SJDBT group and placebo group, resp.). There was no statistically significant group-by-period interaction.

### 3.3. Secondary Outcomes

#### 3.3.1. FAACT and Anorexia VAS

The mean FAACT score and anorexia VAS at each time point are also shown in Table 2. The changes in the FAACT score between baseline and the end of study were −14.13 in SJDBT group and −7.38 in placebo group, but the changes of FAACT score were not significantly different between the two groups (*p* = 0.124). In the within-group analysis, change in SJDBT group and placebo group showed significant difference (*p* < 0.001 and *p* = 0.011, SJDBT group and placebo group, resp.). The changes in the anorexia VAS between baseline and the end of study were 25.78 in SJDBT group and 18.45 in placebo group, but the changes of anorexia VAS were not significantly different between the two groups (*p* = 0.346). In the within-group analysis, change in SJDBT group and placebo group showed significant difference (*p* < 0.001 and *p* = 0.001, SJDBT group and placebo group, resp.). There were no statistically significant group-by-period interactions.

#### 3.3.2. Clinical Laboratory Tests

In between-group analysis, the change of ACTH, cortisol, ghrelin, IL-6, ESR, and CRP between baseline and the end of study did not show statistically significant difference (Table 3). In within-group analysis, all clinical laboratory tests did not show significant difference.

#### 3.3.3. Qi Deficiency and Blood Deficiency Scale

In between-group analysis, the changes of qi deficiency scale and blood deficiency scale between baseline and end of the study did not show statistically significant difference (Table 4). In within-group analysis, qi deficiency scale showed significant changes in both of SJDBT group and placebo group, and blood deficiency scale showed significant change in SJDBT group.

### 3.4. Safety Analysis

At every visit, systolic blood pressure (SBP), diastolic blood pressure (DBP), pulse, AST, ALT, blood urea nitrogen (BUN), and creatinine did not show significant difference between SJDBT group and placebo group (Table 5). In within-group analysis, at 2 weeks, DBP decreased in the placebo group and, at 4 weeks, DBP decreased in the SJDBT group. There was no noteworthy difference between the SJDBT group and placebo group. Some statistically significant changes in DBP were not in the abnormal range.

## 4. Discussion

The present study is double-blind, randomized, placebo-controlled trial that investigated whether SJDBT improves appetite loss measured by FAACT ACS score and anorexia VAS in cancer patients with anorexia after 4 weeks of treatment. However, there was no statistically significant difference between the SJDBT group and placebo group. In addition to appetite loss, general aspect in quality of life measured by FAACT was also assessed after 4 weeks of treatment. But, there was no statistically significant difference between two groups. Otherwise, changes of clinical laboratory tests including ACTH, cortisol, ghrelin, IL-6, ESR, and CRP were unremarkable.

Although there was no statistically significant difference between SJDBT group and placebo group, the changes of FAACT and anorexia VAS in the SJDBT group imply the need for large-scale trial in further studies. Given that the aspect of change of total FAACT score is similar to that of FAACT ACS score, further studies need to focus on not only the anorexia but also overall status of cancer patients.

There was a report that Rikkunshito which is often prescribed for the treatment of anorexia regulates ghrelin secretion and degradation, sensitizes ghrelin receptor, and antagonizes the 5-HT2b/c receptors [19]. However, in the present study, SJDBT did not affect the concentration of ghrelin. It is speculated that these two herbal medicines have different mechanisms on anorexia. The previous study suggested that SJDBT regulates levels of glucagon-like peptide-1 (GLP-1) and peptide tyrosine tyrosine (PYY) which are satiety stimulators in serum [13, 20, 21]. Therefore, in further clinical studies, effects of SJDBT on GLP-1 and PYY need to be investigated.

Although the present study did not show statistically significant difference between SJDBT and placebo, previous preclinical study reported that SJDBT improves cancer-induced weight loss and anorexia in mice [13]. Moreover, in preclinical studies, SJDBT improved immunological function and had antiangiogenic action [22, 23], inhibited cancer cell metastasis by inducing NK cell activity [24], suppressed enlargement of tumor size [25], and alleviated bone marrow suppression by anticancer drug [26]. In clinical studies, SJDBT ameliorated anemia which is a frequent complaint of patients with cancer [27], improved quality of life in patients with cancer receiving chemotherapy [28], and showed immune enhancement effect in patients with advanced pancreatic cancer [29]. Thus, a comprehensive approach and further studies are needed to utilize SJDBT for management of patients with cancer.

Qi deficiency leads to decreased visceral functions and lowered body resistance and blood deficiency is any pathological change characterized by deficiency of blood which fails to nourish organs, tissues, and meridians/channels [30]. In the present study, qi deficiency and blood deficiency were improved in both groups and there was no statistically significant difference.

It has been reported that* Astragali Radix* and ginsenoside Rg1 which is a major component of* Ginseng Radix* have a beneficial effect on anorexia [31, 32]. However, most of the mechanisms of the active compounds of herbal medicine used in anorexia treatments remain unclear [33]. Likewise, molecular mechanisms of compounds in SJDBT also remain unknown and these are major challenges for herbal medicine research [34].

There were some limitations of the present study. The sample size was too small to show statistically significant results. However, the results of the present pilot study will be used for designing the main study. The purpose of this study was to evaluate efficacy of SJDBT for anorexia; thus we only prohibited participants from taking medications that aim to improve appetite, not for other purposes; therefore many participants took herbal medicines which could affect the qi deficiency and blood deficiency for treatment of fatigue or pain, and so on. This has made it difficult to assess the effects of SJDBT on qi deficiency and blood deficiency. It was an inevitable choice for the needs of patients using Korean medicine hospital and for facilitating clinical trial recruitment. For a similar reason, all patients with solid cancer complaining of anorexia were recruited and the number of participants was too small to investigate the difference in the effect of SJDBT and the difference of anorexia according to cancer type. Another limitation of the present study is that the type of chemotherapy drugs was not confirmed and thus we could not confirm the difference of effect of SJDBT according to the type of chemotherapy. This is because the participants received primary treatment of cancer from other medical institutions, and then the study was conducted on participants who visited the trial institution for symptoms management. Although these limitations limit the interpretation of the study results, the present study is meaningful in that such pilot study results are necessary to conduct large-scale, rigorous study in the medical reality in Korea. Rigorous further studies which compensate these defects will be necessary to ascertain the efficacy of SJDBT for anorexia in cancer patient.

## 5. Conclusions

The present pilot study showed the feasibility that SJDBT could be used for management of patients with cancer. Compared to baseline, 4 weeks of SJDBT treatment improved the quality of life assessed by the FAACT and anorexia in patients with cancer. However, there was no statistically significant difference between SJDBT group and placebo group. These findings could be used as preliminary data for further large-scale studies.

## Figures and Tables

**Box 1:**
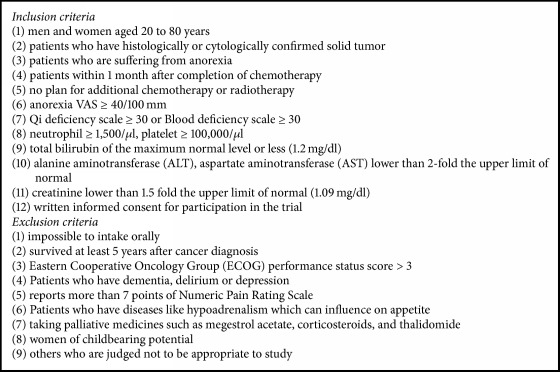
Participants eligibility criteria.

**Table 1:** General characteristics of participants.

Variable	SJDBT group (*n* = 16)	Placebo group (*n* = 16)	*p* value
	Mean ± SD	Mean ± SD	
Age	54.1 ± 8.4	55.1 ± 8.9	0.718
Gender			
Male	1 (6.2%)	1 (6.2%)	1.000
Female	15 (93.8%)	15 (93.8%)	
Anthropometric measurements			
Height, cm	159.2 ± 4.5	156.7 ± 5.4	0.178
Weight, kg	58.4 ± 9.0	59.1 ± 10.2	0.831
BMI	23.0 ± 3.2	24.1 ± 4.5	0.424
FAACT scale	92.6 ± 14.9	92.1 ± 22.9	0.942
FAACT ACS scale	29.5 ± 4.8	30.2 ± 5.5	0.711
Anorexia VAS	59.3 ± 16.8	58.8 ± 19.3	0.937
Systolic blood pressure (SBP), mmHg	110.1 ± 19.0	116.4 ± 12.8	0.275
Diastolic blood pressure (DBP), mmHg	70.6 ± 9.5	75.9 ± 11.7	0.168
Pulse, bpm	71.9 ± 9.7	71.6 ± 9.8	0.929
ACTH	17.8 ± 15.9	19.5 ± 17.9	0.771
Cortisol	10.2 ± 6.5	10.2 ± 5.4	0.995
Ghrelin	1121.2 ± 812.2	978.6 ± 361.4	0.528
Interleukin 6	1.9 ± 1.7	1.8 ± 1.9	0.883
ESR	19.9 ± 10.4	18.7 ± 8.9	0.717
CRP	0.1 ± 0.2	0.1 ± 0.2	0.983
Cancer type			
Breast cancer	3 (18.8%)	4 (25.0%)	0.267
Cervical cancer	2 (12.5%)	0 (0.0%)	
Colon cancer	1 (6.2%)	1 (6.2%)	
Gastric cancer	3 (18.8%)	0 (0.0%)	
Lung cancer	1 (6.2%)	3 (18.8%)	
Thyroid cancer	6 (37.5%)	8 (50.0%)	
Anticancer therapy			
Chemo- and radiotherapy	3 (18.8%)	0 (0.0%)	0.239
Chemotherapy only	3 (18.8%)	4 (25.0%)	
Radiotherapy only	3 (18.8%)	6 (37.5%)	
None	7 (43.8%)	6 (37.5%)	

**Table 2:** Changes from baseline to the end of the study on anorexia.

Variable	Period	SJDBT group (*n* = 16)	Placebo group (*n* = 16)	*p*	Group effect	Period effect
		Mean ± SD	Mean ± SD			
FAACT ACS scale	0 weeks	29.50 ± 4.84	30.19 ± 5.54		0.456	0.028
	2 weeks	33.19 ± 4.86	31.19 ± 4.64			
	4 weeks	34.13 ± 6.03	32.94 ± 6.56			
	Δ(0–4^week^)	−4.63 ± 4.50	−2.75 ± 4.43	0.245		
	ANOVA	Period X group interaction: *p* = 0.602				

FAACT	0 weeks	92.56 ± 14.94	92.06 ± 22.89			
	2 weeks	104.13 ± 17.51	94.44 ± 22.95		0.176	0.118
	4 weeks	106.69 ± 20.00	99.44 ± 25.15			
	Δ(0–4^week^)	−14.13 ± 13.73	−7.38 ± 10.13	0.124		
	ANOVA	Period X group interaction: *p* = 0.661				

Anorexia VAS	0 weeks	59.34 ± 16.78	58.83 ± 19.34			
	2 weeks	39.56 ± 12.46	39.80 ± 23.18		0.600	<0.001
	4 weeks	33.57 ± 19.34	40.38 ± 27.29			
	Δ(0–4^week^)	25.78 ± 24.04	18.45 ± 18.99	0.346		
	ANOVA	Period X group interaction: *p* = 0.730				

FAACT: Functional Assessment of Anorexia Cachexia Therapy; ACS: Anorexia-Cachexia Subscale; VAS: Visual Analogue Scale; *p* represents *p* value of comparison between mean difference of SJDBT group and placebo group.

**Table 3:** Changes from baseline to the end of the study on clinical laboratory tests.

Variable	Period	SJDBT group (*n* = 16)		Placebo group (*n* = 16)		*p*
		Mean ± SD	(*p*_intra_)	Mean ± SD	(*p*_intra_)	
ACTH	0 weeks	17.76 ± 15.94		19.52 ± 17.95		0.771
	4 weeks	15.28 ± 8.94	0.468	15.12 ± 10.58	0.199	0.963
	Δ(0–4^week^)	2.48 ± 13.29		4.40 ± 13.10		0.683

Cortisol	0 weeks	10.22 ± 6.45		10.23 ± 5.44		0.995
	4 weeks	10.19 ± 5.20	0.982	8.60 ± 4.19	0.316	0.350
	Δ(0–4^week^)	0.03 ± 5.25		1.63 ± 6.28		0.441

Ghrelin	0 weeks	1121.19 ± 812.25		978.63 ± 361.44		0.526
	4 weeks	1252.69 ± 753.61	0.036	1255.50 ± 724.46	0.044	0.992
	Δ(0–4^week^)	−131.51 ± 228.54		−276.87 ± 504.58		0.302

IL-6	0 weeks	1.93 ± 1.69		1.83 ± 1.87		0.883
	4 weeks	2.61 ± 2.42	0.213	2.60 ± 4.55	0.412	0.996
	Δ(0–4^week^)	−0.68 ± 2.09		−0.77 ± 3.64		0.934

ESR	0 weeks	19.94 ± 10.36		18.69 ± 8.90		0.717
	4 weeks	20.75 ± 11.46	0.572	18.63 ± 7.47	0.972	0.539
	Δ(0–4^week^)	−0.81 ± 5.62		0.06 ± 7.07		0.701

CRP	0 weeks	0.08 ± 0.17		0.08 ± 0.16		0.983
	4 weeks	0.10 ± 0.27	0.741	0.11 ± 0.20	0.570	0.907
	Δ(0–4^week^)	−0.02 ± 0.28		−0.03 ± 0.22		0.921

ACTH: adrenocorticotropic hormone; IL-6: interleukin 6; ESR: erythrocyte sedimentation rate; CRP: C-reactive protein; (*p*_intra_) represents *p* value of comparison between 0 weeks and 4 weeks within each group; *p* represents *p* value of comparison between SJDBT group and placebo group in each period.

**Table 4:** Changes from baseline to the end of the study on qi and blood deficiency.

Variable	Period	SJDBT group (*n* = 16)		Placebo group (*n* = 16)		*p*
		Mean ± SD	(*p*_intra_)	Mean ± SD	(*p*_intra_)	
Qi	0 weeks	56.25 ± 9.90		58.56 ± 12.01		0.557
	4 weeks	38.75 ± 14.54	0.002	39.19 ± 14.31	<0.001	0.932
	Δ(0–4^week^)	17.50 ± 18.18		19.38 ± 15.71		0.757

Blood	0 weeks	47.56 ± 13.16		47.94 ± 16.63		0.944
	4 weeks	36.63 ± 15.31	0.034	36.81 ± 18.57	0.076	0.975
	Δ(0–4^week^)	10.94 ± 18.76		11.13 ± 23.34		0.980

(*p*_intra_) represents *p* value of comparison between 0 weeks and 4 weeks within each group; *p* represents *p* value of comparison between SJDBT group and placebo group in each period.

**Table 5:** The result for safety analysis.

Variable	Period	SJDBT group (*n* = 16)		Placebo group (*n* = 16)		*p*
		Mean ± SD	(*p*_intra_)	Mean ± SD	(*p*_intra_)	
SBP, mmHg	0 weeks	110.06 ± 18.99		116.44 ± 12.83		0.275
	2 weeks	111.94 ± 16.38	0.471	114.38 ± 16.04	0.445	0.674
	4 weeks	112.50 ± 17.75	0.397	116.81 ± 12.30	0.893	0.431

DBP, mmHg	0 weeks	70.63 ± 9.45		75.94 ± 11.68		0.168
	2 weeks	73.81 ± 9.03	0.140	70.13 ± 11.48	0.038	0.321
	4 weeks	75.31 ± 12.47	0.047	76.19 ± 10.06	0.922	0.829

Pulse, bpm	0 weeks	71.94 ± 9.74		71.63 ± 9.82		0.929
	2 weeks	73.38 ± 11.98	0.477	73.81 ± 6.24	0.216	0.898
	4 weeks	75.94 ± 9.94	0.128	70.38 ± 7.88	0.460	0.090

AST, IU/L	0 weeks	20.94 ± 6.81		21.38 ± 5.44		0.842
	4 weeks	24.94 ± 14.09	0.157	23.06 ± 7.13	0.217	0.638

ALT, IU/L	0 weeks	17.31 ± 9.41		21.63 ± 11.53		0.256
	4 weeks	20.13 ± 13.39	0.210	22.50 ± 10.92	0.609	0.587

BUN, mg/dL	0 weeks	12.19 ± 2.85		13.95 ± 3.80		0.149
	4 weeks	13.44 ± 2.84	0.138	13.38 ± 3.07	0.489	0.953

Cre, mg/dL	0 weeks	0.75 ± 0.10		0.80 ± 0.11		0.242
	4 weeks	0.76 ± 0.08	0.513	0.79 ± 0.09	0.779	0.320

SBP: systolic blood pressure; DBP: diastolic blood pressure; AST: aspartate aminotransferase; ALT: alanine transferase; BUN: blood urea nitrogen; Cre: creatinine; (*p*_intra_) represents *p* value of comparison between 0 weeks and *n* weeks within each group; *p* represents *p* value of comparison between SJDBT group and placebo group in each period.

## Abbreviations

SJDBT: Sipjeondaebo-tang
FAACT: Functional Assessment of Anorexia and Cachexia Treatment
ACS: Anorexia/Cachexia Subscale
FACT-G: Functional Assessment of Cancer Therapy-General
VAS: Visual Analogue Scale
BMI: Body mass index
ACTH: Adrenocorticotropic hormone
ALT: Alanine aminotransferase
AST: Aspartate aminotransferase
ECOG: Eastern Cooperative Oncology Group
ISEE: Institute of Safety, Efficacy and Effectiveness Evaluation for Korean Medicine
CRO: Contract Research Organization
SOPs: Standard Operating Procedures
ESR: Erythrocyte sedimentation rate
CRP: c-reactive protein
CRF: Case report form
ITT: Intention-to-treat
SBP: Systolic blood pressure
DBP: Diastolic blood pressure
BUN: Blood urea nitrogen
GLP-1: Glucagon-like peptide-1
PYY: Peptide tyrosine tyrosine.
