# Corrigendum to “Efficacy and Safety of Sipjeondaebo-Tang for Anorexia in Patients with Cancer: A Pilot, Randomized, Double-Blind, Placebo-Controlled Trial”

**DOI:** 10.1155/2018/6162106

**Published:** 2018-07-30

**Authors:** Chunhoo Cheon, Jeong-Eun Yoo, Hwa-Seung Yoo, Chong-Kwan Cho, Sohyeon Kang, Mia Kim, Bo-Hyoung Jang, Yong-Cheol Shin, Seong-Gyu Ko

**Affiliations:** ^1^Department of Preventive Medicine, Korean Medical College, Kyung Hee University, Seoul, Republic of Korea; ^2^Dunsan Korean Medicine Hospital of Daejeon University, Daejeon, Republic of Korea; ^3^Department of Cardiovascular and Neurologic Disease (Stroke Center), College of Korean Medicine, Kyung Hee University, Seoul, Republic of Korea

In the article titled “Efficacy and Safety of Sipjeondaebo-Tang for Anorexia in Patients with Cancer: A Pilot, Randomized, Double-Blind, Placebo-Controlled Trial” [1], there was an error in the conclusion of the Abstract where the text reading “Sipjeondaebo-tang appears to have potential benefit for anorexia management in patients with cancer. Further large-scale studies are needed to ensure the efficacy” should be corrected to “In the present study, Sipjeondaebo-tang did not show a significant effect on anorexia in patients with cancer. Further large-scale studies which compensate for the limitations of this study are needed to assess the efficacy”.

This error in the reporting was brought to the attention of the journal by Professor Edzard Ernst, Emeritus Professor of Complementary Medicine, University of Exeter. The journal and the authors apologize for this misreporting of the conclusions.
